# Pro‐Inflammatory Biomarkers in Stable Versus Acutely Decompensated Heart Failure With Preserved Ejection Fraction

**DOI:** 10.1161/JAHA.117.007385

**Published:** 2018-04-12

**Authors:** Abraham Abernethy, Sadi Raza, Jie‐Lena Sun, Kevin J. Anstrom, Russell Tracy, Johannes Steiner, Peter VanBuren, Martin M. LeWinter

**Affiliations:** ^1^ The Cardiology Unit University of Vermont Burlington VT; ^2^ Department of Molecular Physiology and Biophysics University of Vermont Burlington VT; ^3^ Department of Pathology University of Vermont Burlington VT; ^4^ Duke Clinical Research Institute Durham NC

**Keywords:** biomarker, decompensated heart failure, diastolic dysfunction, diastolic heart failure, ejection fraction, heart failure, pro‐inflammatory biomarkers, Biomarkers, Cell Signalling/Signal Transduction, Growth Factors/Cytokines, Pathophysiology, Heart Failure

## Abstract

**Background:**

Underlying inflammation has been increasingly recognized in heart failure with a preserved ejection fraction (HFpEF). In this study we tested the hypothesis that pro‐inflammatory biomarkers are elevated in patients with acutely decompensated HFpEF (AD‐HFpEF) compared with patients with stable HFpEF (S‐HFpEF).

**Methods and Results:**

Using a post hoc analysis the serum biomarkers tumor necrosis factor‐alpha, high‐sensitivity C‐reactive protein interleukin 6 and pentraxin 3 (PTX3) and clinical, demographic, echocardiographic‐Doppler and clinical outcomes data were analyzed in HFpEF patients enrolled in NHLBI Heart Failure Research Network clinical trials which enrolled patients with either AD‐HFpEF or S‐HFpEF. Compared to S‐HFpEF, AD‐HFpEF patients had higher levels of PTX3 (3.08 ng/mL versus 1.27 ng/mL,* P*<0.0001), interleukin‐6 (4.14 pg/mL versus 1.71 pg/mL,* P*<0.0001), tumor necrosis factor‐alpha (11.54 pg/mL versus 8.62 pg/mL,* P*=0.0015), and high‐sensitivity C‐reactive protein (11.90 mg/dL versus 3.42 mg/dL,* P*<0.0001). Moreover, high‐sensitivity C‐reactive protein, interleukin‐6 and PTX3 levels were significantly higher in AD‐HFpEF compared with S‐HFpEF patients admitted for decompensated HF within the previous year. PTX3 was positively correlated with left atrial volume index (*r*=0.41, *P*=0.0017) and left ventricular mass (*r*=0.26, *P*=0.0415), while tumor necrosis factor‐alpha was inversely correlated with E/A ratio (*r*=−0.31, *P*=0.0395).

**Conclusions:**

Levels of pro‐inflammatory biomarkers are strikingly higher in AD‐HFpEF compared with S‐HFpEF patients. PTX3 and tumor necrosis factor‐alpha are correlated with echocardiographic‐Doppler evidence of diastolic dysfunction. Taken together these data support the concept that a heightened pro‐inflammatory state has a pathophysiologic role in the development of AD‐HFpEF.

**Clinical Perspective**

**What Is New**

- Elevated levels of pro‐inflammatory serum biomarkers are well‐described in heart failure with reduced ejection fraction (HFrEF), but much less is known in patients with heart failure with a preserved ejection fraction (HFpEF), especially when they are decompensated.
- In this retrospective analysis, we report serum values of interleukin‐6, tumor necrosis factor‐alpha, h‐sensitivity C‐reactive protein, and pentraxin 3 in patients with stable and acutely decompensated HFpEF.
- In stable HFpEF, levels of tumor necrosis factor‐alpha and high‐sensitvity C‐reactive protein were >1 SD above the mean for subjects without prevalent cardiovascular disease, whereas interleukin‐6 and pentraxin 3 were below this cut‐off.
- All 4 biomarkers were strikingly and significantly elevated in decompensated versus stable HFpEF; further analysis suggests that these biomarker levels rise in conjunction with decompensation.

**What Are the Clinical Implications?**

- These results are consistent with the hypotheses that chronic inflammation plays a role in the pathophysiology of HFpEF and that heightened inflammation may occur with decompensation; whether changes in inflammation precede decompensation will require prospective studies.
- If additional studies support these hypotheses, they may provide a rationale for targeted anti‐inflammatory therapy in HFpEF.

## Introduction

Inflammation, oxidative stress, and extracellular matrix remodeling have been long considered to play a key role in the pathophysiology of heart failure (HF).^1^, ^2^, ^3^, ^4^, ^5^, ^6^, ^7^, ^8^, ^9^ A number of studies have revealed a significant correlation between elevations in blood levels of pro‐inflammatory biomarkers such as tumor necrosis factor alpha (TNF‐α), C‐reactive protein (CRP),interleukin 6 (IL‐6), and HF severity and prognosis.^10^, ^11^, ^12^, ^13^, ^14^, ^15^, ^16^, ^17^, ^18^, ^19^, ^20^ Recently, pentraxin 3 (PTX3), an acute‐phase reactant member of the CRP super family expressed by a variety of cell types in response to various inflammatory stimuli^21^ and thought to be a marker of vascular inflammation, was found to facilitate risk stratification of HF patients.^22^, ^23^, ^24^, ^25^, ^26^ Other pro‐inflammatory biomarkers that may have prognostic value in HF include galectin‐3 and ST2.^27^, ^28^

It has been proposed that a heightened pro‐inflammatory state may play a role in the progression of HF, including acute decompensations.^1^, ^2^, ^3^, ^4^, ^5^, ^6^, ^7^, ^8^, ^9^ Impairment of nitric oxide bioavailability due to oxidative stress, leading to impaired cyclic guanosine monophosphate‐protein kinase G signaling^29^ is one potential mechanism underlying the link between inflammation and HF progression. Another proposed mechanism involves an imbalance of collagen production and breakdown mediated by matrix metalloproteinases and tissue inhibitors of metalloproteinases in the extracellular matrix. As such, biomarkers of collagen turnover may also provide prognostic value.^30^

Most information in regard to inflammation and HF has come from patients with HF and a reduced left ventricular ejection fraction (HFrEF). Recently, there has been an effort to better characterize biomarkers in HF with preserved EF (HFpEF)^2^, ^9^, ^31^, ^32^). While inflammation plays a role in both HFpEF and HFrEF,^33^ there appear to be distinct differences in their pathogenesis.^2^, ^34^, ^35^, ^36^ We are unaware of any data in regard to whether more intense inflammation plays a role in *acutely decompensated* HFpEF (AD‐HFpEF). We hypothesized that heightened inflammation is a component of the pathogenesis of acute decompensation in HFpEF. As an initial, preliminary test of this hypothesis, in the present study we sought to determine if selected pro‐inflammatory biomarkers are elevated in patients with AD‐HFpEF compared with patients with stable HFpEF (S‐HFpEF).

## Methods

The data, analytic methods, and study materials will not be made available to other researchers for purposes of reproducing the results or replicating the procedures.

We measured IL‐6, TNF‐α, and hs‐CRP and PTX3 in age and sex‐matched cohorts of HFpEF patients from 3 prospective, placebo‐controlled, randomized clinical trials conducted by the NHLBI Heart Failure Research Network, RELAX (Effect of Phosphodiesterase‐5 Inhibition on Exercise Capacity and Clinical Status in Heart Failure with Preserved Ejection Fraction),^37^ DOSE (Diuretic Strategies in Patients With Acute Decompensated Heart Failure)^38^ and ROSE (Renal Optimization Strategies Evaluation).^39^ RELAX included S‐HFpEF patients while DOSE and ROSE included acutely decompensated HF (ADHF) patients with both HFrEF and HFpEF. IL‐6, TNF‐α, and hs‐CRP were selected because of the long‐standing experience with their use in HF^10^, ^11^, ^12^, ^13^, ^14^, ^15^, ^16^, ^17^, ^18^, ^19^, ^20^ and PTX3 because of its association with vascular inflammation^21^ and ability to risk‐stratify HF patients. Each trial was approved by the Institutional Review Board at each participating site, and all patients provided written informed consent.

### Patient Selection

HFpEF patients were selected from the aforementioned trials. The RELAX trial tested the effect of the phosphodiesterase‐5 inhibitor sildenafil on exercise capacity and clinical status in comparison with placebo in S‐HFpEF patients. DOSE examined responses to 2 different diuretic strategies in hospitalized patients with ADHF. The primary end points of DOSE were a symptom score and changes in renal function. The in‐patient treatment strategy was maintained for 72 hours. Clinical outcomes were determined for 72 hours and follow‐up status was assessed 30 and 60 days after completion. ROSE was a trial of hospitalized patients with ADHF and renal dysfunction (estimated glomerular filtration rate,^40^ 15 to 60 mL/min per 1.73 m^2^) comparing 72 hours of low‐dose dopamine or low‐dose nesiritide to a standardized diuretic strategy. The dual end points of ROSE were cumulative urine volume and change in serum cystatin C from enrollment to 72 hours. Clinical status was assessed 30 and 60 days after completion.

Patients were eligible for enrollment in RELAX if they had chronic symptomatic HF with EF ≥0.50, elevated N‐terminal brain‐type natriuretic peptide (NT‐proBNP), elevated invasively‐measured left ventricular filling pressures or diastolic dysfunction by echocardiography‐Doppler examination, and reduced peak VO2. For the purpose of the current analysis, all RELAX patients were considered eligible unless they had been hospitalized with ADHF within the previous 6 months. Prior hospitalizations in RELAX patients were identified based on review of the patients’ records by the local principal investigators.

Patients selected from DOSE and ROSE constituted the AD‐HFpEF group. Patients were eligible for the current analysis if they had EF ≥0.50 and would have been eligible for RELAX had they not been hospitalized for ADHF. The DOSE and ROSE exclusion criteria included acute co‐morbidities that could independently affect pro‐inflammatory biomarkers, including infections and other active conditions associated with heightened inflammation (eg, connective tissue diseases, cancer, acute coronary syndromes). By design, both S‐HFpEF and AD‐HFpEF cohorts were split evenly between males and females.

### Biomarkers

hs‐CRP, IL‐6,TNF‐α and PTX3 were measured at baseline exams by the NHLBI Heart Failure Research Network Biomarker Core Laboratory at the University of Vermont using commercially available kits (hs‐CRP, Siemens, Indianapolis; IL‐6, Meso Scale Discovery, Gaithersbury, MD; TNF‐α, EMD Millipore, Billerica, MA; PTX3, R&D systems, Minneapolis, MN). Samples were shipped on dry ice and stored at −80°C until analysis at the Biomarker Core Laboratory.

In addition to determining whether there are differences in pro‐inflammatory biomarkers in AD‐HFpEF versus S‐HFpEF, we also tested whether biomarker levels are associated with echocardiographic‐Doppler abnormalities of left ventricular diastolic function in all comers and are predictive of clinical outcomes in AD‐HFpEF. In the DOSE and ROSE trials short‐term clinical outcomes included urine volume, change in cystatin‐C, change in creatinine, change in NT‐proBNP, and change in weight over the 72 hours after randomization. In addition, post‐discharge clinical status with respect to survival and re‐hospitalizations was determined by telephone call 60 days after randomization for each trial.

### Statistical Analysis

Baseline patient characteristics were compared between AD‐HFpEF and S‐HFpEF patients. Continuous variables were reported as median (25th, 75th quartiles) and compared using Wilcoxon rank‐sum tests. Categorical variables are reported as frequencies and percentages, and were compared using the Pearson chi‐square test or the Fisher exact test. The distributions of the 4 biomarkers (PTX3, hsCRP, IL‐6, and TNF‐α) were compared between AD‐HFpEF and S‐HFpEF using box plots with Wilcoxon rank sum tests.

For the S‐HFpEF group, the Spearman correlation coefficient was used to assess correlations between biomarkers and diastolic function indexes at baseline and 24 weeks. Diastolic function indexes tested were LA volume index, E/A ratio, medial E/e′, lateral E/e′ and relative wall thickness.

For the AD‐HFpEF group, unadjusted and age‐adjusted Cox proportional hazards regression models were used to assess the association between biomarkers and death or HF hospitalization at 60 days. Unadjusted and age‐adjusted linear regression models were used to assess the association between biomarkers and 72‐hour end points. The linearity assumption was assessed by a likelihood ratio test comparing the linear fit to the fit of a restricted cubic spline transformation. Linear spline transformations were applied to the biomarkers that did not satisfy the linearity assumption. The proportional hazards assumption was tested for the Cox models by plotting the Schoenfeld residuals by time and then testing for a non‐zero slope. As there were no violations we report the single hazard ratio as an average over time. The adjusted models were only adjusted for age because of the small sample size.

For PTX3, hsCRP, and IL‐6 there were less than 2% missing values. For TNF‐α there were ≈10% missing values. Complete case analyses were conducted. All statistical tests were two‐sided, and the criterion for statistical significance was a *P*‐value less than 0.05. Statistical analyses were performed using statistical software (version 9.3, SAS Institute Inc, Cary, NC).

## Results

Baseline clinical characteristics of the 2 groups are shown in Table 1. Compared to S‐HFpEF, AD‐HFpEF patients had lower systolic blood pressure (119 versus 122 mm Hg, *P*=0.029), were more likely to have jugular venous pressure ≥8 cm (94.4% versus 45.0%, *P*<0.001), peripheral edema (69.7% versus 14.5%, *P*<0.001), and to report symptoms of orthopnea (85.1% versus 57.7%, *P*<0.001). 94.2% of the AD‐HFpEF cohort had NYHA class III or IV symptoms at baseline while, by design, the S‐HFpEF cohort consisted exclusively of patients with NYHA class II and III symptoms. The AD‐HFpEF cohort was more likely to have been admitted for heart failure within the past year (61.8% versus 34.9%, *P*<0.001), to have atrial fibrillation and/or flutter (70.5% versus 50.6%, *P*≤0.001), diabetes mellitus (DM) (61.5% versus 34.9%, *P*<0.001), chronic obstructive pulmonary disease (COPD) (34.6% versus 19.3%, *P*=0.028) and anemia (71.8% versus 30.1%, *P*<0.001). The AD‐HFpEF cohort was less likely to be treated with an angiotensin converting enzyme inhibitor (ACEi) or angiotensin receptor blocker (38.5% versus 63.9%, *P*=0.001), but was more likely to be receiving an aldosterone receptor antagonist (25.6% versus 6.0%, *P*<0.001) or a loop diuretic (97.4% versus 77.1%, *P*<0.001). The AD‐HFpEF cohort had worse renal function than the S‐HFpEF group (serum creatinine 1.6 mg/dL versus 1.2 mg/dL, *P*<0.001; cystatin C 1.7 mg/L versus 1.4 mg/L, *P*<0.001; eGFR^40^ 40.0 mL/min per 1.73 m^2^ versus 53.5 mL/min per 1.73 m^2^, *P*<0.001) and higher levels of NT‐pro BNP (3146 pg/mL versus 648.1 pg/mL, *P*<0.001).

**Table 1:** Baseline Characteristics of Patients With Stable and Acutely Decompensated HFpEF

Characteristic	S‐HFpEF (n=83)	AD‐HFpEF (n=78)	Overall (n=161)	*P* Value
Demographics				
Age, years: n, median (25th–75th)	83, 72 (65–79)	78, 73 (65–79)	161, 72 (65–79)	0.970
Male sex	43/83 (51.8%)	39/78 (50.0%)	82/161 (50.9%)	0.819
Race (% white)	74/83 (89.2%)	62/78 (79.5%)	136/161 (84.5%)	0.091
Weight, kg (median, 25th–75th)	93.2 (80.5–103.6)	95.0 (79.1–112.3)	93.2 (80.5–108.2)	0.535
Body mass index, (median, 25th–75th)	32.3 (27.4–37.1)	33.6 (28.5–37.9)	32.9 (27.9–37.5)	0.163
Ejection fraction: n, median (25th–75th)	83, 62.0 (58.0–66.0)	78, 55.0 (55.0–61.0)	161, 60.0 (55.0–65.0)	<0.001
Systolic blood pressure, mm Hg: n, median (25th–75th)	83, 122 (113–137)	78, 119 (107–131)	161, 122 (112–135)	0.029
Heart rate, beats/min: n, median (25th–75th)	83, 69 (62–80)	78, 72 (65–84)	161, 72 (62–80)	0.073
JVP≥8 cm	36/80 (45.0%)	68/72 (94.4%)	104/152 (68.4%)	<0.001
Edema≥2	12/83 (14.5%)	53/76 (69.7%)	65/159 (40.9%)	<0.001
Comorbidities				
Hospitalization for heart failure in past year	29/83 (34.9%)	47/76 (61.8%)	76/159 (47.8%)	<0.001
Hypertension	65/83 (78.3%)	69/78 (88.5%)	134/161 (83.2%)	0.085
Ischemia as cause of HF	33/83 (39.8%)	37/78 (47.4%)	70/161 (43.5%)	0.326
Atrial fibrillation/flutter	42/83 (50.6%)	55/78 (70.5%)	97/161 (60.2%)	0.010
Diabetes mellitus	29/83 (34.9%)	48/78 (61.5%)	77/161 (47.8%)	<0.001
Orthopnea	45/78 (57.7%)	63/74 (85.1%)	108/152 (71.1%)	<0.001
COPD	16/83 (19.3%)	27/78 (34.6%)	43/161 (26.7%)	0.028
NYHA class				<0.001
II	40/83 (48.2%)	4/69 (5.8%)	44/152 (28.9%)	
III	43/83 (51.8%)	50/69 (72.5%)	93/152 (61.2%)	
IV	0/83 (0.0%)	15/69 (21.7%)	15/152 (9.9%)	
Baseline anemia	25/83 (30.1%)	56/78 (71.8%)	81/161 (50.3%)	<0.001
Medications at enrollment				
ACE inhibitor or ARB	53/83 (63.9%)	30/78 (38.5%)	83/161 (51.6%)	0.001
Beta blockers	61/83 (73.5%)	64/78 (82.1%)	125/161 (77.6%)	0.193
Aldosterone antagonist	5/83 (6.0%)	20/78 (25.6%)	25/161 (15.5%)	<0.001
Any diuretic	70/83 (84.3%)	76/78 (97.4%)	146/161 (90.7%)	0.004
Loop diuretic	64/83 (77.1%)	76/78 (97.4%)	140/161 (87.0%)	<0.001
Calcium channel blocker	26/83 (31.3%)	25/78 (32.1%)	51/161 (31.7%)	0.921
Statin	54/83 (65.1%)	49/78 (62.8%)	103/161 (64.0%)	0.767
Laboratory values				
Sodium, mg/L: n, median (25th–75th)	75, 140 (138–142)	78, 139 (137–142)	153, 140 (137–142)	0.127
Blood urea nitrogen, mg/dL: n, median (25th–75th)	68, 25.1 (18.7–35.0)	78, 33.5 (26.0–49.0)	146, 30.0 (22.0–44.0)	<0.001
Creatinine, mg/dL: n, median (25th–75th)	83, 1.2 (0.9–1.5)	78, 1.6 (1.3–1.9)	161, 1.4 (1.1–1.8)	<0.001
NT‐pro BNP, pg/mL: n, Median (25th–75th)	83, 648.1 (352.9–1334)	78, 3146 (1583–5747)	161, 1482 (596.0–3172)	<0.001
eGFR, mL/min; n, median (25th–75th)	83, 53.5 (39.0–71.4)	78, 40.0 (30.0–48.9)	161, 45.6 (34.5–57.7)	<0.001
Baseline core lab cystatin C value (mg/L): n, median (25th–75th)	83, 1.4 (1.0–1.8)	78, 1.7 (1.4–2.1)	161, 1.6 (1.2–2.0)	<0.001

*P*‐values for continuous variables: Wilcoxon Rank‐Sum test. *P*‐values for Categorical variables: Pearson chi‐square test or Fisher's exact test. ACE indicates angiotensin converting enzyme; ARB, angiotensin receptor blocker; COPD, chronic obstructive pulmonary disease; eGFR, estimated glomerular filtration rate; HFpEF, heart failure with a preserved ejection fraction; JVP, jugular venous pressure; NT‐pro BNP, N‐terminal pro b‐type natriuretic peptide; NYHA, New York Heart Association.

Pro‐inflammatory biomarker results are shown in FigureA. Compared with the S‐HFpEF cohort, the AD‐HFpEF cohort had higher levels of IL‐6 (4.14 pg/mL versus 1.71 pg/mL, *P*<0.001), TNF‐α (11.54 pg/mL versus 8.62 pg/mL, *P*=0.002), hs‐CRP (11.90 mg/dL versus 3.42 mg/dL, *P*<0.001) and PTX3 (3.08 ng/mL versus 1.27 ng/mL, *P*<0.001). In the CHS (Cardiovascular Health Study),^41^ the geometric mean±SD for PTX3 in subjects without prevalent cardiovascular disease (CVD) was 1.64±1.8 ng/mL. The median value of 1.27 ng/mL in S‐HFpEF is within one standard deviation of the mean for subjects without CVD. Based on the Health‐ABC (Health, Aging and Body Composition) Study,^42^ the median (25th–75th percentile) value for IL‐6 in subjects without prevalent CVD was 1.6 (1.1–2.4) pg/mL. The median value of 1.71 pg/mL in S‐HFpEF is well within this range. Based on Health‐ABC,^41^ the median (25th–75th percentile) value for TNF‐α in subjects without prevalent CVD is 3.0 (2.3–3.8) pg/mL. The median value of 8.62 pg/mL in S‐HFpEF is well above this range. Finally, in CHS^43^ the median (25th–75th percentile) value for hs‐CRP in subjects without prevalent CVD was 1.76 (0.88–3.10) mg/dL. The median value of 3.42 mg/dL in S‐HFpEF is modestly above this range. All of the median biomarker values in AD‐HFpEF were clearly above the range in subjects without prevalent CVD.

**Figure 1:**
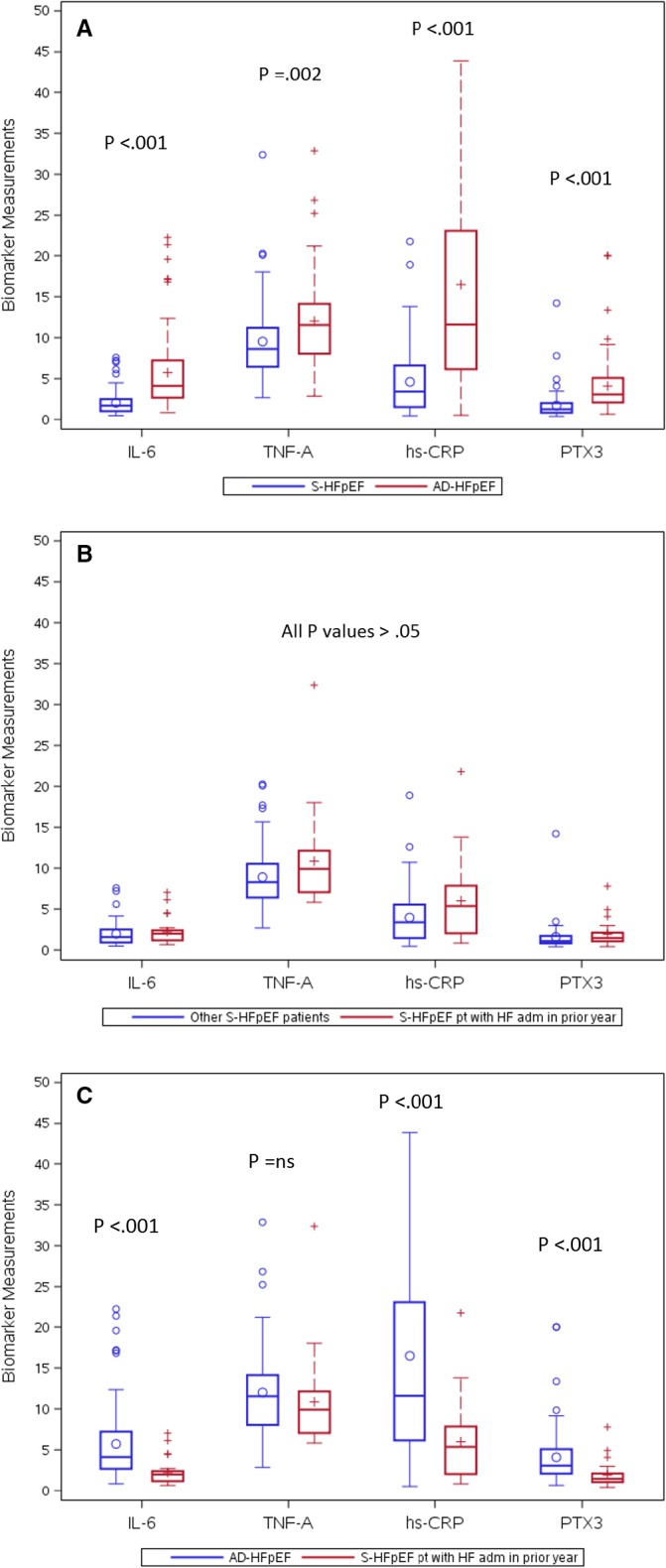
A, Box and whisker plots of median (solid lines within boxes) and interquartile ranges for pro‐inflammatory biomarkers in S‐HFpEF (RELAX) versus AD‐HFpEF (DOSE and ROSE) patients. Whiskers=ranges from bottom to top 25% of data values, excluding outliers. The latter are shown individually above the whiskers. Small circles and+signs within boxes=mean values. B, Box and whisker plots of pro‐inflammatory biomarkers for S‐HFpEF patients with a HF admission in the prior year versus other S‐HFpEF patients. C, Box and whisker plots of pro‐inflammatory biomarkers for all AD‐HFpEF patients versus S‐HFpEF patients with a HF admission during the prior year. AD‐HFpEF indicates acutely decompensated heart failure with a preserved ejection fraction; DOSE, Diuretic Strategies in Patients With Acute Decompensated Heart Failure; HFpEF, heart failure with a preserved ejection fraction; hs‐CRP indicates high‐sensitivity C‐reactive protein; IL‐6, interleukin 6; PTX3, pentraxin 3; RELAX, Effect of Phosphodiesterase‐5 Inhibition on Exercise Capacity and Clinical Status in Heart Failure with Preserved Ejection Fraction; ROSE, Renal Optimization Strategies Evaluation; TNF‐A, tumor necrosis factor‐α (see text).

Because the S‐HFpEF and AD‐HFpEF groups differed with respect to the incidence of atrial fibrillation, DM, and COPD we performed a separate analysis in which the biomarker results were adjusted for these 3 variables (Table 2). The results indicate that for PTX3, IL‐6, and TNF‐α 10% to 20% of the differences between S‐HFpEF and AD‐HFpEF can be explained by adjusting for all 3 conditions. For hs‐CRP the adjustment had minimal impact on the results.

**Table 2:** Mean Biomarker Differences Between AD‐HFpEF and S‐HFpEF Using Unadjusted Linear Regression Model and Linear Regression Model Adjusted for Atrial Fibrillation, DM, and COPD

Outcomes	Models	Estimate	Standard Error	*P* Value	Total N
PTX3	Unadjusted	2.38	0.44	<0.001	161
	Adjusted	2.05	0.47	<0.001	161
IL‐6	Unadjusted	3.67	0.55	<0.001	158
	Adjusted	3.30	0.59	<0.001	158
TNF‐A	Unadjusted	2.48	0.86	0.004	145
	Adjusted	1.98	0.91	0.032	145
hs‐CRP	Unadjusted	13.83	2.65	<0.001	157
	Adjusted	14.05	2.89	<0.001	157

hs‐CRP indicates high‐sensitivity C‐reactive protein; IL‐6, interleukin 6; PTX3, pentraxin 3; TNF‐A, tumor necrosis factor‐α. AD‐HFpEF indicates acutely decompensated heart failure with a preserved ejection fraction; COPD, chronic obstructive pulmonary disease; DM, diabetes mellitus; S‐HFpEF, stable heart failure with a preserved ejection fraction.

Blood samples obtained after discharge from the hospital were not available in the AD‐HFpEF group. Therefore, to help determine whether elevated pro‐inflammatory biomarkers are a transient finding associated with decompensation in this group or are persistently elevated, we divided the S‐HFpEF patients into groups with and without an ADHF admission within the previous year (FigureB). Although there were nominally increased biomarker levels in the group with a recent admission for ADHF, there were no statistically significant differences in the S‐HFpEF cohort with recent admission compared to the S‐HFpEF cohort without recent ADHF admission (IL‐6 2.00 pg/mL versus 1.57 pg/mL, *P*=0.160; TNF‐α 9.92 pg/mL versus 8.28 pg/mL, *P*=0.128; hs‐CRP 5.36 mg/dL versus 3.38 mg/dL, *P*=0.063; PTX3 1.46 ng/mL versus 1.08 ng/mL, *P*=0.057. As shown in FigureC, compared with the S‐HFpEF group with recent ADHF admissions the AD‐HFpEF cohort had significantly higher levels of IL‐6 (4.14 pg/mL versus 2.00 pg/mL, *P*<0.001), hs‐CRP (11.90 mg/dL versus 5.36 mg/dL, *P*<0.001) and PTX3 (3.08 ng/mL versus 1.46 ng/mL, *P*<0.001), but there was no statistically significant difference in TNF‐α (11.54 pg/mL versus 9.92 pg/mL, *P*=0.202). The latter results suggest that PTX3, IL‐6, and hs‐CRP are not persistently elevated in patients with AD‐HFpEF, while TNF‐α may remain elevated after admission for ADHF or alternatively may decline more gradually.

We also sought correlations between pro‐inflammatory biomarkers and echocardiography‐Doppler derived measurements of diastolic function at baseline for the S‐HFpEF patients. As shown in Table 3, PTX3 was found to be positively correlated with left atrial volume index (*P*=0.002, Spearman coefficient=0.41) and left ventricular mass (*P*=0.042, Spearman coefficient=0.26), while elevated TNF‐α was found to be inversely correlated with E/A ratio (*P*=0.040, Spearman coefficient=−0.31). IL‐6 and hs‐CRP were not significantly correlated with any measures of diastolic function**.**

**Table 3:** Correlations Between Biomarkers and Diastolic Function Indexes at Baseline in the RELAX Cohort

	Spearman Correlation Coefficients *P* Values Number of Observations					
	LA Volume Index	E/A Ratio	Medial E/e′ (m/sec)	Lateral E/e′ (m/sec)	Relative Wall Thickness	LV Mass
PTX3	0.41	0.18	0.02	−0.16	0.08	0.26
	0.002	0.196	0.872	0.165	0.523	0.042
	57	53	73	73	60	60
IL‐6	0.11	−0.11	0.18	0.22	0.23	0.05
	0.438	0.434	0.134	0.066	0.084	0.691
	57	52	72	72	60	60
TNF‐α	0.06	−0.31	0.17	0.15	0.14	0.16
	0.672	0.040	0.176	0.238	0.305	0.244
	52	46	65	65	54	54
hs‐CRP	−0.12	−0.10	0.07	0.07	0.10	0.00
	0.400	0.468	0.567	0.569	0.435	0.987
	55	52	71	71	59	59

hs‐CRP indicates high sensitivity C reactive protein; IL‐6, interleukin 6; LA, left atrial; LV, left ventricular; PTX3, pentraxin 3; RELAX, Effect of Phosphodiesterase‐5 Inhibition on Exercise Capacity and Clinical Status in Heart Failure with Preserved Ejection Fraction; TNF‐A, tumor necrosis factor‐α.

In the AD‐HFpEF cohort, biomarkers were evaluated for their association with short‐term clinical outcomes and re‐hospitalization or death within 60 days after admission. There were no statistically significant associations between short‐term outcomes (urine volume, change in cystatin‐C, change in creatinine, change in NT‐proBNP, change in weight) and biomarker levels. Re‐admission or death within 60 days after discharge occurred in 13 of 68 AD‐HFpEF patients. In an unadjusted model, none of the biomarkers were significantly associated with re‐admission or death. In the age‐adjusted model, PTX3 had a HR of 0.863 (95% confidence interval0.662–1.123, *P*=0.273), IL‐6 had a HR of 1.097 (95% confidence interval 1.003–1.201, *P*=0.043), TNF‐α had a HR of 1.014 (95% confidence interval 0.925–1.112, *P*=0.768), and hs‐CRP had a HR of 1.015 (95% confidence interval 0.993–1.037, *P*=0.182). Thus, the only biomarker that exhibited a statistically significant association with 60 day death or heart failure re‐hospitalization was IL‐6 using the age‐adjusted model.

## Discussion

Over the past 2 decades, there has been an evolution in our understanding of the complex pathophysiology of HFpEF and associated diastolic dysfunction (reviewed in 44, 45, 46). Major risk factors for HFpEF include hypertension, type 2 diabetes mellitus and insulin resistance and obesity (all elements of the metabolic syndrome), obstructive sleep apnea, chronic kidney disease and aging. Concentric left ventricular remodeling and associated increased diastolic chamber stiffness and slowed relaxation are the rule, while increased systolic chamber and arterial stiffness are common. At the level of the myocardium, altered calcium handling,^47^ increased collagen content and cross‐linking^48^, ^49^ and alterations in phosphorylation of contractile proteins^50^ and titin^29^, ^49^ cause increased passive stiffness and slowed and incomplete myofilament relaxation. These advances in understanding the pathophysiology of HFpEF have led to attempts to more precisely phenotype the syndrome^44^ in an effort to better guide treatment.

As discussed earlier, in HFrEF a pro‐inflammatory state that appears to contribute to its pathophysiology has been recognized for many years. More recently, the contribution of a pro‐inflammatory state to the pathophysiology of HFpEF has been emphasized.^6^, ^8^, ^17^, ^23^, ^31^, ^34^, ^35^, ^36^, ^45^ In patients with ADHF, biomarkers reflecting activation of the renin‐angiotensin aldosterone system (RAAS) (plasma renin activity, aldosterone levels), oxidative stress (uric acid) and collagen synthesis (N‐terminal procollagen III) appear to be elevated to a similar extent in HFrEF and HFpEF.^33^ Previous studies have shown that the RAAS and adrenergic nervous system activation are capable of triggering inflammation in the heart.^51^, ^52^ The hypothesis that inflammation initiated in vascular endothelium plays a key role in the pathogenesis of HFpEF has recently been advanced, especially in conjunction with obesity and other elements of the metabolic syndrome.^6^, ^35^ This derangement has been proposed to result in impaired nitric oxide availability and protein kinase G signaling with subsequent reduced phosphorylation of protein kinase G/protein kinase A sites on titin.^29^ The latter, along with increased phosphorylation of protein kinase C sites^48^ increase titin's stiffness and its contribution to total passive myocardial stiffness. These same pro‐inflammatory signals may contribute to increased collagen content and fibrosis.^6^, ^30^, ^33^, ^35^

In light of these findings, we hypothesized that worsening of an underlying pro‐inflammatory state contributes to clinical decompensation in HFpEF. In the present study, as *a first step* in testing this hypothesis, we measured levels of a panel of pro‐inflammatory biomarkers in patients with S‐HFpEF compared with patients with AD‐HFpEF.

In S‐HFpEF, our results show that of the pro‐inflammatory biomarkers measured, PTX3 and IL‐6 levels were in a range comparable to those in adults without prevalent CVD, hs‐CRP was modestly elevated and TNF‐α was substantially elevated. We are unaware of any comparable measurements in patients with well‐characterized *stable* HFpEF. These results suggest that pro‐inflammatory pathways are activated in a selective fashion in S‐HFpEF. Compared to S‐HFpEF, AD‐HFpEF patients had consistent and striking elevations in hsCRP, IL‐6, TNF‐α, and PTX3 (FigureA), supporting our underlying hypothesis. Based on our adjusted analysis (Table 2), differences in the prevalence of atrial fibrillation, DM and COPD between the S‐HFpEF and AD‐HFpEF groups accounted for only a modest proportion of the biomarker differences.

Because serial samples were not available, we do not know if AD‐HFpEF patients as a class have persistently higher levels of pro‐inflammatory biomarkers as opposed to a transient rise occurring in association with decompensation. To shed light on this issue, we separated the S‐HFpEF patients into those who had and those who had not had an acute decompensation in the prior year and compared them to each other and with the AD‐HFpEF group. There were no statistically significant differences in pro‐inflammatory biomarker levels between S‐HFpEF patients with and without recent admissions for ADHF, although the trends we observed toward higher values in patients with recent admissions may indicate that our sample size was too small. With the exception of TNF‐α, levels of pro‐inflammatory biomarkers were greater in AD‐HFpEF patients than in S‐HFpEF patients with recent ADHF admissions, with highly significant *P* values. Taken together, these results suggest that PTX3, IL‐6, and hsCRP rise transiently either before or in parallel with HFpEF decompensation and subsequently tend to decrease gradually. In the absence of blood samples acquired before decompensation, it is not possible to state whether these rises precede decompensation, which would make a stronger case for a cause and effect relationship. It is not clear whether TNF‐α truly behaves differently or if the relatively small numbers of patients limited our power to detect a difference.

Because there may be a mechanistic link between inflammation and diastolic dysfunction in HFpEF, we tested for correlations between the levels of the pro‐inflammatory biomarkers we assayed and echocardiographic‐Doppler measures. We found a highly significant correlation between PTX3 and left atrial volume index (*r*=0.41, *P*=0.002) and weaker correlations between PTX3 and LV mass (*r*=0.26, *P*=0.042) and TNF‐α and E/A ratio (*r*=−0.31, *P*=0.040). Left atrial volume index is a relatively robust measure of diastolic function as it is less acutely load‐sensitive than various Doppler derived indexes. The correlation between PTX3 and left atrial volume may be related to the fact that this relatively recently described biomarker has a strong association with myocardial fibrosis and, consistent with the endothelial inflammation hypothesis, is strongly expressed by vascular endothelium.^21^, ^22^, ^23^, ^53^ Whether PTX3 truly has a specific pathophysiologic role in HF is not known. However, it has previously been correlated with diastolic function and is produced in the coronary circulation in patients with HF.^23^

In addition to testing our primary hypothesis, to evaluate whether levels of pro‐inflammatory biomarkers could serve as a prognostic tool to identify AD‐HFpEF patients at increased risk for future clinical events, we related the levels to combined re‐hospitalizations and deaths over the 60 day period after the index admission. The only statistically predictive biomarker we identified was IL‐6, which was just significant (HR 1.097, *P*=0.043).

### Limitations

There are several limitations to our study. Most importantly, the number of patients was relatively small and, as with most post hoc analyses, not specifically powered to test our hypothesis. In addition, because of the small sample size and the relatively low event rate in the AD‐HFpEF group we only adjusted for age using the Cox model to test for associations between biomarkers and outcomes. Thus, our results should be considered hypothesis‐generating, rather than definitive. A second limitation is the baseline clinical differences in the S‐HFpEF and AD‐HFpEF cohorts. The AD‐HFpEF cohort was by definition sicker in terms of NYHA Class, and more likely to have co‐morbid atrial fibrillation, DM, renal dysfunction and COPD. Our adjusted analysis taking atrial fibrillation, DM and COPD into account indicates that they are responsible for only a modest percentage of the biomarker differences. There were also significant differences in ACEi, angiotensin receptor blocker, aldosterone blocker and loop diuretic use. These differences could have independently influenced biomarker levels. Echocardiograms were not obtained at baseline as part of the DOSE and ROSE protocols. As a result, we cannot be certain that LVEF did not change between the time of the most recent echocardiographic study, which was used to classify patients as HFpEF or HFrEF, and the time of enrollment. As noted earlier, exclusion criteria for DOSE and ROSE included a number of co‐morbidities that could have contributed to a recent EF change had they been present. Moreover, Dunlay et al^54^ systematically studied EF changes in HFpEF patients. They found that EF declined by an average of 5.8% over 5 years. Thus, we believe it is unlikely that patients were misclassified based on interval changes in EF. Finally, as noted earlier serial blood samples were not available after recovery from decompensation in the AD‐HFpEF group, which would have allowed a more rigorous test of whether elevated levels are transient.

### Conclusions and Future Directions

Levels of the 4 pro‐inflammatory biomarkers we measured are selectively elevated in patients with S‐HFpEF, but all were strikingly higher in AD‐HFpEF compared with S‐HFpEF. S‐HFpEF patients with recent ADHF admissions tended to have higher levels of pro‐inflammatory biomarkers than S‐HFpEF patients without recent admissions and, with the exception of TNF‐α, levels of pro‐inflammatory biomarkers were significantly higher in AD‐HFpEF patients than S‐HFpEF patients with recent ADHF admissions. Taken together, these results suggest that PTX3, IL‐6, and hsCRP rise transiently (either before or in parallel) with HFpEF decompensation and subsequently tend to decrease gradually.

At present, it is not definitively known whether inflammation is merely associated with HFpEF or is a central factor in its pathophysiology and/or etiology. Our results are supportive of the hypothesis that increases in pro‐inflammatory biomarkers are closely connected with decompensation in HFpEF and, in the case of PTX3, may also be directly linked to diastolic dysfunction. To more definitively test a mechanistic role for a heightened pro‐inflammatory state as a driver of decompensation in HFpEF will require larger numbers of patients and, ideally, an attempt to measure biomarkers prospectively, before decompensation. This might be done by taking measurements at predetermined intervals in stable HFpEF patients and determining if rising levels predict decompensation. If these results were positive, they could provide insights into which patients might benefit from early intervention to reduce the need for hospitalization as well as those who in whom trials of anti‐inflammatory regimens directed at specific pro‐inflammatory signaling pathways might be warranted.

## Sources of Funding

This work was supported by grants from the National Heart, Lung, and Blood Institute (Coordinating Center: U10 HL084904; Regional Clinical Centers: U01 HL084861, U10 HL110312, U109 HL110337, U01 HL084889, U01 HL084890, U01 HL084891, U10 HL110342, U10 HL110262, U01 HL084931, U10 HL110297, U10 HL110302, U10 HL110309, U10 HL110336, and U10 HL110338).

## Disclosures

None.
